# *In vitro* high affinity α-synuclein binding sites for the amyloid imaging agent PIB are not matched by binding to Lewy bodies in postmortem human brain^1^

**DOI:** 10.1111/j.1471-4159.2008.05245.x

**Published:** 2008-05

**Authors:** Liang Ye, Ana Velasco, Graham Fraser, Thomas G Beach, Lucia Sue, Tracy Osredkar, Vincenzo Libri, Maria Grazia Spillantini, Michel Goedert, Andrew Lockhart

**Affiliations:** * GlaxoSmithKline, Clinical Science and Technology, Neurology DM New Frontiers Science Park, Harlow, UK; † MRC Laboratory of Molecular Biology Cambridge, UK; ‡ Civin Laboratory for Neuropathology, Sun Health Research Institute Sun City, Arizona, USA; § Cambridge Centre for Brain Repair, Robinson Way Cambridge, UK

**Keywords:** α-synuclein, Alzheimer's disease, amyloid, imaging, Lewy body, Parkinson's disease

## Abstract

Amyloid containing deposits are a defining neuropathological feature of a wide range of dementias and movement disorders. The positron emission tomography tracer PIB (Pittsburgh Compound-B, 2-[4′-(methylamino)phenyl]-6-hydroxybenzothiazole) was developed to target senile plaques, an amyloid containing pathological hallmark of Alzheimer's disease, formed from the amyloid-β peptide. Despite the fact that PIB was developed from the pan-amyloid staining dye thioflavin T, no detailed characterisation of its interaction with other amyloid structures has been reported. In this study, we demonstrate the presence of a high affinity binding site (*K*_d_∼4 nM) for benzothiazole derivatives, including [3H]-PIB, on α-synuclein (AS) filaments generated *in vitro*, and further characterise this binding site through the use of radioligand displacement assays employing 4-*N*-methylamino-4′-hydroxystilbene (SB13) (*K*_i_ = 87 nM) and 2-(1-{6-[(2-fluoroethyl(methyl)amino]-2-naphthyl}ethylidene)malononitrile (FDDNP) (*K*_i_ = 210 nM). Despite the presence of a high-affinity binding site on AS filaments, no discernible interaction of [3H]-PIB was detected with amygdala sections from Parkinson's disease cases containing frequent AS-immunoreactive Lewy bodies and related neurities. These findings suggest that the density and/or accessibility of AS binding sites *in vivo* are significantly less than those associated with amyloid-β peptide lesions. Lewy bodies pathology is therefore unlikely to contribute significantly to the retention of PIB in positron emission tomography imaging studies.

*J. Neurochem.* (2008) **105,** 1428–1437.

A common neuropathological finding associated with both normal ageing and a range of dementias is the presence of senile plaques (SPs) formed from amyloid-beta (Aβ) peptides (Braak and Braak 1991; Bouras *et al.* 1994; Tsuboi and Dickson 2005; Ballard *et al.* 2006). Their occurrence in sufficient density and distribution, together with neurofibrillary tangles (NFTs), in histological sections of postmortem cerebral cortex is considered diagnostic of Alzheimer's disease (AD) (Mirra *et al.* 1991; Goedert and Spillantini 2006). The ability to definitively confirm AD diagnosis intra vitam would represent a major clinical advance and one such technique that has seen major recent advances is the field of amyloid imaging using positron emission tomography (PET) (Nordberg 2007).

A series of PET radiotracer chemotypes are currently under investigation in AD patient populations and include [18F]-FDDNP (2-(1-{6-[(2-fluoroethyl(methyl)amino]-2-naphthyl}ethylidene)malononitrile) (Shoghi-Jadid *et al.* 2002), Pittsburgh Compound-B (2-[4′-(methylamino)phenyl]-6-hydroxybenzothiazole) ([11C]-PIB) (Klunk *et al.* 2004), [11C]-4-*N*-methylamino-4′-hydroxystilbene (SB13) (Verhoeff *et al.* 2004) and [11C]-BF-227 (Kudo *et al.* 2007). *In vitro* data suggests that these ligands have a high affinity for Aβ only when it is the form of amyloid fibrils (Klunk *et al.* 2003; Lockhart *et al.* 2005). Consistent with this observation all of the ligands, with the exception of [18F]-FDDNP, display broadly similar cortical uptake in the frontal, parietal, temporal and occipital lobes of AD patients. [18F]-FDDNP additionally displayed an increased uptake in the medial temporal lobe which is suggestive of an enhanced interaction with NFTs (Shoghi-Jadid *et al.* 2002; Small *et al.* 2006), even though there is a paucity of *in vitro* work supporting this interpretation (Lockhart 2006).

Although the imaging tracers were developed to bind to Aβ fibrils their precise molecular target is not the Aβ peptide sequence *per se* but rather the generic β-pleated sheet structure that forms the amyloid folds (Fowler *et al.* 2007). Amyloid structures are therefore not restricted to Aβ containing lesions but can also be formed by a range of other proteins, including some that are also common in neurodegenerative disease, such as tau and α-synuclein (AS), which give rise to NFTs and Lewy bodies (LBs) respectively (Serpell *et al.* 2000; Berriman *et al.* 2003). A number of imaging studies are now reporting data from patient populations with diseases other than AD, including frontotemporal lobar degeneration (Engler *et al.* 2008; Rabinovici *et al.* 2007) and dementia with Lewy bodies (DLB) (Bacskai *et al.* 2007; Johansson *et al.* 2007; Rowe *et al.* 2007), in which NFTs and LBs, respectively, form a significant part of the pathological load. It is therefore critical that a comprehensive amyloid binding profile is assembled for current PET tracers using experimental conditions that are relevant to the low nanomolar tissue concentrations that are attained during imaging scans (Mathis *et al.* 2004; Rowe *et al.* 2007).

Lewy bodies and Lewy neurites are filamentous inclusions that form in the cytoplasm of susceptible neurons and are primarily associated with Parkinson's disease (PD) and DLB (Goedert 2001). However, neuropathological studies have suggested that up to 60% of AD cases also exhibit significant LB pathology (Kotzbauer *et al.* 2001). Factors leading to the appearance, heterogeneous morphology and neuroanatomical location of LBs are only poorly understood (Goedert 2001; Kotzbauer *et al.* 2001). The inclusions typically consist of a central core and an outer halo, with the former containing both amorphous material and filamentous structures. AS was first identified as a component of LBs and Lewy neurites (Spillantini *et al.* 1997) and then immunoelectron microscopy, coupled with biochemical analyses, demonstrated that it forms the filamentous structures associated with these lesions (Baba *et al.* 1998; Spillantini *et al.* 1998). Confirming their amyloid credentials, LBs can be visualised in tissue sections using the dyes thioflavin S and Congo Red (Dickson 2005), although staining with the latter dye can be relatively weak (Smid *et al.* 2006).

A number of previous *in vitro* studies interrogating the specificity of the amyloid tracers for Aβ-containing lesions produced a somewhat confusing picture because of the use of different experimental techniques and concentrations of ligand. For example, initial studies using 100–1000 nM PIB demonstrated that the ligand could bind to Aβ lesions (both classical (i.e. neuritic and cored) plaques and cerebrovascular amyloid) as well as NFTs (Klunk *et al.* 2003; Mathis *et al.* 2004). However, binding assays using low nanomolar concentrations of ligand coupled with brain homogenates from a range of neuropathological disorders appeared to suggest a high degree of specificity for Aβ lesions (Klunk *et al.* 2003). The question of binding specificity was recently re-addressed using low nanomolar concentrations of tritium-labelled PIB ([3H]-PIB), coupled with high-resolution autoradiography (Lockhart *et al.* 2007). This study demonstrated that at concentrations of PIB that are directly relevant for *in vivo* imaging, the ligand labelled all types of Aβ-containing lesions (diffuse plaques, classical plaques and cerebrovascular amyloid) with high affinity. Although additional labelling of NFTs was also observed, the intensity of labelling was less than that associated with the Aβ-containing pathologies. These findings suggest that rather than being a specific marker of AD, or specific types of Aβ-containing lesions in AD, PIB is a non-specific marker of Aβ peptide-related cerebral amyloidosis.

There have been a limited number of reports describing the interaction of amyloid ligands with AS filaments and/or LBs. Homogenate binding assays using the PIB-related compound [3H]-2-(4′-methylaminophenyl)benzothiazole (BTA-1) failed to detect any significant binding in one DLB case (Klunk *et al.* 2003). In support of this finding, a more detailed investigation has indicated that although high-affinity binding sites were present on AS filaments generated *in vitro*, LB pathology could not be labelled in tissue sections from DLB brains or in DLB brain tissue homogenate binding assays (Fodero-Tavoletti *et al.* 2007). In contrast, LB pathology is routinely detected using compounds structurally related to PIB, such as thioflavins (Conway *et al.* 2000), and has also been identified using novel amyloid ligands, such as FDDNP (Smid *et al.* 2006). A clear disconnect exists between these studies and we believe that it derives in large part from the mixture of different assay techniques used (radioligand binding assays vs. fluorescence microscopy) that differ in sensitivity and tissue preparation.

We therefore undertook the present study, where we have used a small panel of amyloid imaging agents to characterise their interactions with AS filaments generated *in vitro*. In addition, autoradiography on frozen human amygdala (AMY) sections containing LB pathology was carried out by using concentrations of [3H]-PIB relevant for *in vivo* imaging. The findings reported here provide fundamental information on the nature of the interactions of amyloid agents with AS filaments and the associated LB pathology.

## Materials and methods

### Compound names and sources

**Fig. 1:**
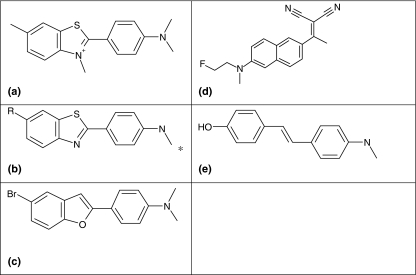
Chemical structures of compounds used in the study. A, Thioflavin T; B, BTA, R = HO (PIB)/CH_3_ (6Me-BTA-1)/H (BTA-1); C, BF1; D, FDDNP; E, SB13. *Denotes position of radiolabel on [3H]-Me-BTA-1 and [3H]-PIB. Full compound nomenclatures are provided in the Materials and methods section entitled Compound names and sources.

The structures of the compounds used in this study are shown in Fig. 1 and are as follows; 2-[4′-(methylamino)phenyl]-6-methylbenzothiazole (6-Me-BTA-1); 2-[4′-(methylamino)phenyl]-6-hydroxylbenzothiazole (6-OH-BTA-1/PIB); SB13; FDDNP; and 5-bromo-2-(4-dimethylaminophenyl)benzofuran (BF1). Thioflavin T was obtained from Merck Chemicals (Nottingham, UK). The remaining compounds were custom synthesised and confirmed for purity by reverse phase HPLC, one-dimensional NMR and mass spectrometry. Radiolabelled 2-[4′-([3H]methylamino)phenyl]-6-methylbenzothiazole ([3H]Me-BTA-1) (84 Ci/mmol, 1 mCi/mL) and *N*-methyl-[3H]2-(4′-methylaminophenyl)-6-hydroxylbenzothiazole ([3H]-PIB) (71 Ci/mmol, 1 mCi/mL), were custom synthesised by Amersham Biosciences (Little Chalfont, UK). All other chemicals were from Sigma-Aldrich (Gillingham, UK).

### Preparation of α-synuclein and formation of filaments

The expression and purification of recombinant AS were as described (Jakes *et al.*, 1994; Masuda *et al.* 2006a). Fibrillation reactions were performed in a shaking incubator at 37°C using 400 μM AS in 30 mM 3-[*N*-morpholino] propanesulphonic acid, pH 7.2 [3-[*N*-morpholino] propanesulphonic acid assay buffer (MAB)], containing 0.02% sodium azide, for up to 10 days (Serpell *et al.* 2000).

### Gel analysis of α-synuclein assemblies

Samples (20 μL) were removed at days 1, 2, 5 and 10 from the assembly reactions, mixed with 80 μL MAB and centrifuged at 50 000 *g* for 30 min at 20°C. The resulting high-speed supernatant (HSS) was removed and the high-speed pellet (HSP) re-suspended in 100 μL MAB. HSS and HSP were mixed with sodium dodecyl sulphate (SDS)-loading buffer and separated by SDS–polyacrylamide gel electrophoresis using NuPage 4–12% Bis–Tris gels (Invitrogen, Paisley, UK). The gels were calibrated using Precision Plus Protein Prestained Standards (BioRad, Hemel Hempsted, UK), stained for total protein using Simply Blue Safestain (Invitrogen) and the images captured on a ChemiGenius Bio Imaging System (Syngene, Cambridge, UK).

### Thioflavin T fluorescence binding assay

Assays were performed in MAB using a fixed concentration of AS (1 μM) and either a fixed (25 μM) or varying concentrations of ligand (0–25 μM) as noted in the Results section. Reactions were incubated for 1 h at 20°C before reading on an Ultra Evolution 384 plate reader (Tecan, Reading, UK) employing a 450–505 nm filter pair. All data points were performed at least in quadruplicate and were analysed using Grafit (Erithacus Software Limited, Horley, UK) to obtain *K*_d_ values using the single site ligand binding module.

### Radioligand binding assay of α-synuclein

Assays were performed as described (Lockhart *et al.* 2005) using a fixed concentration of AS (200 nM) and a range of [3H]-Me-BTA-1 or [3H]-PIB concentrations (40, 16, 6.4, 2.56, 1.026, 0.4096, 0.16384 and 0.065536 nM) with the following modifications. Reactions were performed in MAB for 1 h and non-specific binding was determined using 50 μM Thioflavin T. Data were analysed using Grafit to obtain the apparent dissociation constant (*K*_d_) and the maximum number of binding sites (*B*_max_) using the single-site ligand binding module.

### Radioligand competition assays of α-synuclein

Assays employed a fixed concentration of AS (200 nM), and [3H]Me-BTA-1 (4 nM) as described previously (Lockhart *et al.* 2005), except that the reactions were performed in MAB. Reactions were performed in MAB for 1 h and non-specific binding was determined using 50 μM Thioflavin T. All data points were performed in triplicate, and the specific binding signal in the absence of competitor defined a fractional binding of 1. Data were analysed using Grafit to obtain IC_50_ values using 4-parameter curve fits. *K*_i_ values were calculated using the Cheng–Prusoff equation (Cheng and Prusoff 1973): *K*_i_ = IC_50_/(1 + [L]/*K*_d_, where [L] was the concentration of [3H] Me-BTA-1 used in the assay (4 nM) and the *K*_d_ was from the radioligand binding assay for each batch of AS filaments.

### [3H]-PIB autoradiography

Human brain tissue was obtained from the Brain Donation Program at Sun Health Research Institute. Two series of four consecutive cryosections (8–10 μm thickness) of frozen AMY from each of four cases of PD were mounted onto statically charged (plus) glass slides prior to airtight storage with desiccants at −80°C. The first (Slide 1) and third (Slide 3) sections in each series were used for histochemical analysis. The second and the fourth sections were used in the radioligand binding studies defining total (0.5 or 2 nM [3H]-PIB) and non-displaceable (0.5 or 2 nM [3H]-PIB plus 10 μM BTA-1) binding signals respectively.

Slides were thawed for 10 min at room temperature (25°C) prior to incubation in phosphate-buffered saline (PBS) buffer containing 10% v/v ethanol, pH 7.2) for 15 min at 25°C. Slides were then incubated with either [3H]-PIB or [3H]-PIB plus BTA-1 for 1 h at 25°C and the reactions terminated by consecutive 30 s incubations in (PBS buffer containing 10% v/v ethanol, pH 7.2 (×2) and distilled water (all at 4°C). The slides were dried under a cold air stream and then apposed with Hyperfilm-3H (RPN535B: early availability batch; Amersham Biosciences).

Sections (Slide 1) were removed from the freezer and immediately immersed in 70% ethanol for 2 min. Endogenous peroxidase was then suppressed by immersion in 1% hydrogen peroxide for 30 min. This was followed by antigen exposure with 20% formic acid for 1 min. After additional washes, sections were incubated overnight in primary antibody (mouse anti-human AS, monoclonal antibody clone LB509; Zymed, San Francisco, CA, USA), diluted 1 : 1000. Sections were then sequentially incubated in biotinylated anti-mouse IgG, avidin–biotin peroxidase complex (ABC; Vector Elite, Burlingame, CA, USA) and 3,3′-diaminobenzidine (1 mg/mL), dehydrated and mounted in Permount. For thioflavin S staining, sections (Slide 3) were removed from the freezer and immediately immersed for 30 min in 4% freshly prepared formaldehyde, pH 7.4. Following washes in PBS and partial drying at 25°C, the sections were defatted in 1 : 1 chloroform/ethanol for 1 h, washed briefly twice in 100% ethanol and then hydrated through graded alcohols to water. Sections were then stained in 0.1% thioflavin S for 10 min, differentiated rapidly in 80% ethanol and coverslipped with Apathy's medium.

## Results

### Formation and characterisation of α-synuclein filaments

α-Synuclein can be expressed recombinantly and assembled into filaments that share many features of those isolated from LBs. The relative ease of production of recombinant AS has made it an attractive material for biochemical studies (Crowther *et al.* 1998; Serpell *et al.* 2000), structural analysis (Serpell *et al.* 2000) and inhibitor screening (Masuda *et al.* 2006b; Luk *et al.* 2007). In order to ensure that AS filaments could be assembled in a form that was suitable for characterisation, their properties were further assessed using ultracentrifugation and thioflavin T fluorescence.

**Fig. 2:**
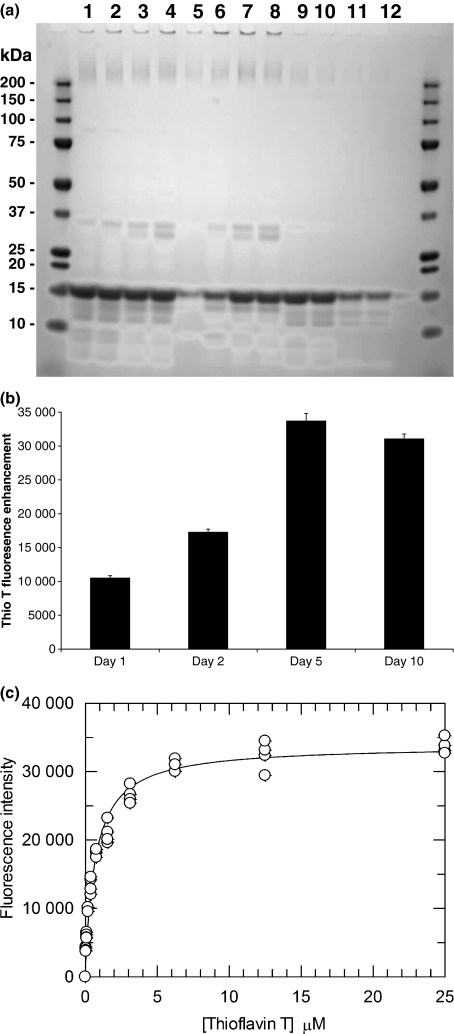
Characterisation of AS fibrils. (a) Representative SDS–polyacrylamide gel electrophoresis, stained for total protein, showing time course of polymerisation reaction. Lanes 1–4 Uncentrifuged reaction mixture; 5–8, HSP; 9–12, HSS. The monomeric molecular weight of AS is ∼15 kDa. (b) Temporal enhancement of Thioflavin T fluorescence demonstrating maximal binding at day 5. (c) Saturation binding of Thioflavin T to day 5 AS fibrils.

α-Synuclein filaments were produced using a previously established method (Serpell *et al.* 2000). The assay mixtures were sampled after one (day 1), two (day 2), five (day 5) and 10 (day 10) days. Separation of filaments from soluble non-amyloid forms of AS was performed using ultracentrifugation and the resulting HSS and HSP were analysed by SDS–polyacrylamide gel electrophoresis (Fig. 2a). Gel analysis demonstrated a time-dependent loss of soluble AS from the HSS and its corresponding appearance in the HSP. The incorporation of AS into the HSP plateaued by day 5.

The amyloid content of the reaction mixtures was monitored by thioflavin T fluorescence (Fig. 2b). Characteristic of amyloid, the AS preparations produced an enhancement in dye fluorescence, which increased with time of incubation and peaked at day 5, with no further change evident by day 10. A binding constant for thioflavin T was derived for the day 5 filaments by using a fixed concentration of polymer and varying concentrations of the fluorophore (Fig. 2c). The resulting binding isotherm was consistent with the presence of a single class of binding sites with a *K*_d_ value of 588 ± 2 nM. Based on the data from these screening assays AS filaments formed after 5 days incubation were selected for further characterisation using radioligand binding assays. Negative stain electron microscopy confirmed the filamentous nature of these preparations (data not shown).

### Radioligand binding assays

**Fig. 3:**
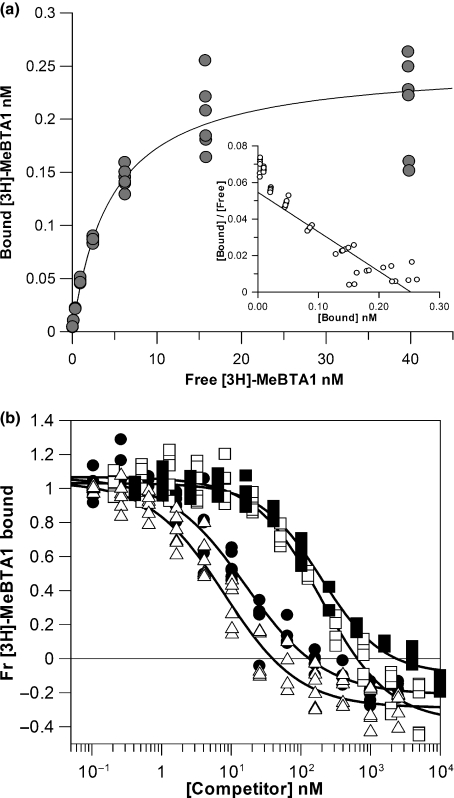
(a) Binding isotherm for the ligand [^3^H]Me-BTA-1 with day 5 AS fibrils. Scatchard analysis is shown in inset. The *K*_d_ and *B*_max_ values derived from this analysis were 4.62 and 0.25 nM respectively. (b) Radioligand competition assays. Dose–response curves showing the fractional binding of [^3^H]Me-BTA-1 to day 5 AS fibrils in the presence of competitor ligands; △, BF1; ▄, FDDNP; •, PIB; □, SB13.

A preparation of tritium-labelled [3H]-Me-BTA1 (Fig. 1) was used to characterise the AS filaments, as the radiotracer has been extensively investigated with Aβ fibril preparations (Lockhart *et al.* 2005; Ye *et al.* 2005). Typical data from one of the six individual batches of day 5 AS filaments analysed is shown in Fig. 3a. The fitted binding isotherm was consistent with the presence of a single class of binding site and produced *K*_d_ and *B*_max_ values of 4.62 and 0.25 nM respectively. Composite data from the six batches demonstrated good consistency, with *K*_d_ and *B*_max_ values of 4.09 ± 0.82 and 0.22 ± 0.02 nM respectively (Table 1). As the concentration of polymer used in the assays was 200 nM the *B*_max_ value corresponds to one radioligand binding site per ∼900 AS monomers. A tritium-labelled preparation of 2-[4′-(methylamino)phenyl]-6-hydroxylbenzothiazole ([3H]-PIB) was also evaluated with one batch of AS filaments and produced values more or less identical to that obtained with [3H]-Me-BTA1, with *K*_d_ and *B*_max_ values of 4.16 and 0.16 nM respectively (data not shown).

**Table 1:** Summary of binding constant data from six batches of AS filaments

	*K*_d_ and *K*_i_ values (nM)				
	
	Me-BTA1	PIB	FDDNP	SB13	BF1
AS	4.09 ± 0.82	16.5 ± 4.36 (4.16^#^)	210.17 ± 81.38	87 ± 20.96	4.78 ± 0.43*
Aβ	4.2^a^	25^b^, 4.7^c^	42^b^	6^d^	3^a^

Comparative literature values for *in vitro* generated Aβ fibrils are shown; ^#^ and * indicates the data from one or four batches of AS filaments respectively.

a Lockhart *et al.* 2005;

b Ye *et al.* 2005;

c Mathis *et al.* 2003;

d Ono *et al.* 2002.

The interaction of additional ligands with AS filaments was assessed using a previously described radioligand displacement assay format utilising [3H]-Me-BTA1 (Lockhart *et al.* 2005) (Fig. 3b). All of the competitor ligands examined (PIB, SB13, BF1 and FDDNP) displayed a dose-dependent displacement of the radioligand from the AS filaments. The derived *K*_i_ values are shown in Table 1. BF1 (4.78 ± 0.43 nM) was the most potent competitor ligand and displayed a binding affinity comparable with [3H]-Me-BTA1. Unlabelled PIB (16.5 ± 4.36 nM) was around fourfold weaker than BF1 in this assay format and this contrasts with the higher affinity interaction determined with the [3H]-PIB preparation. Both SB13 (87 ± 20.96 nM) and FDDNP (210.17 ± 81.38 nM) demonstrated notably lower affinity binding interactions which were ∼20- and ∼50-fold weaker than what was observed for BF1.

In summary, these results are consistent with the presence of at least one class of ligand binding site on the AS filaments generated *in vitro*. This site has a fairly broad ligand binding specificity and occurs at low density along the polymer. It is worth noting that in contrast to our previous studies using Aβ fibrils (Lockhart *et al.* 2005; Ye *et al.* 2005), ethanol was omitted from the buffer as it was found to interfere with the assay, possibly through destabilisation of the AS polymers. Ethanol is required to maintain the solubility of these agents at the higher concentrations (up to 10 μM) used in the radioligand displacement assays (Lockhart *et al.* 2005) and its omission may, in part, explain the increased variability in the *K*_i_ values of the weakest displacers, SB13 and FDDNP.

### *Ex vivo* autoradiography of [3H]-PIB with human brain tissue containing LB pathology

**Fig. 4:**
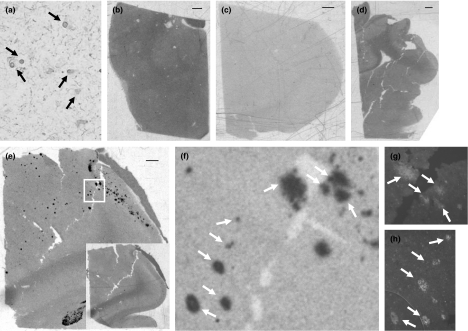
Analysis of amygdala sections from PD cases. (a) Representative image of section from case A3 showing the extensive immunoreactivity to α-synuclein associated with this case. Lewy body pathology is highlighted with black arrows. (b–d) Low resolution autoradiographic images of entire amygdala sections from cases A1–A3. Following labelling with 2 nM [3H]-PIB and film exposure of 8 weeks no discrete labelling puncta were observed. (e) Case A4 displayed significant punctate labelling with [3H]-PIB. The inset to (e) demonstrates that the radiolabel is fully displaceable in the presence of a cold competitor ligand (10 μM BTA-1). (f) High power view of boxed area in E together with Thioflavin S staining of adjacent section (g and h) indicating the presence of significant senile plaque pathology. Arrows show correspondence of diffuse plaques (g) and classical plaques (h) with [3H]-PIB labelled features. Scale bar: 1 mm.

The presence of binding sites on the AS filaments raised the possibility that *in vivo* LB pathology might contribute to the uptake and retention of imaging agents such as PIB. In order to explore this hypothesis further, autoradiography was used to evaluate the binding of [3H]-PIB to LBs and Lewy neurites in AMY sections from four individuals with pathologically confirmed PD (cases A1–A4, Table 2). AMY sections were chosen, as this brain region is often severely affected by LB pathology (Braak *et al.* 1994) and all of the sections used in the study had high densities of LBs and Lewy neurite pathologies (Fig. 4a). The concentrations of [3H]-PIB utilised in this part of the study were chosen to reflect the low nanomolar concentrations of ligand typically attained during imaging studies (Mathis *et al.* 2004; Rowe *et al.* 2007).

**Table 2:** Pathology scoring of AMY sections and case summaries associated with [3H]-PIB autoradiography study

Case number	DP	CP	NFT	CAA	LB	Clinical diagnosis	ApoE status
A1	−	−	−	−	+++	PDD	3/3
A2	−	−	−	−	+++	PDD	2/3
A3	−	−	−	−	+++	PDD	3/4
A4	+	++	−	−	++	PDD	2/3

DP, diffuse plaques; CP, classical plaques; NFT, neurofibrillary tangles; CAA, cerebrovascular amyloid angiopathy deposits; LB, Lewy bodies; PDD, Parkinson's disease with dementia; ApoE, apolipoprotein E; AMY, amygdala. Pathology scores: +++, frequent; ++, moderate; +, sparse; −, zero.

Two sets of sections were labelled with tracer concentrations of [3H]-PIB (0.5 and 2 nM) and apposed to film for 8 weeks. Three of the four cases analysed showed no detectable association of the radiolabel with discrete structures within the neuropil (Fig. 4b–d). Case A4, however, demonstrated a discrete punctate labelling with [3H]-PIB (Fig. 4e). The vast majority of the radiolabel associated with this case was fully displaceable in the presence of unlabelled competitor ligand (BTA-1, 10 μM) demonstrating that the labelling was specific (Fig. 4e inset).

The nature of the pathological features associated with the radiolabelled areas was investigated further through analysis of the adjacent sections stained with thioflavin S or an anti-AS antibody. Thioflavin S revealed a staining pattern that was consistent, based on morphology (staining intensity, shape and size), with the presence of diffuse plaques (Fig. 4g) and classical plaques (Fig. 4h). These were distributed across the section, but were most concentrated in a band running down from the upper, right hand corner. A high degree of correlation between SP pathology and the radiolabelled features was demonstrated (Fig. 4f–h).

Staining with the anti-AS antibody indicated that LB pathology was largely confined to a narrow band of lesions in the upper right corner of the section. Unfortunately, at this resolution, and because of the far greater size of the thioflavin S-positive SPs also occupying the same area, it was not possible to ascertain if LBs corresponded to any of the [3H]-PIB-labelled lesions. Micro-autoradiography techniques may provide the necessary higher resolution to dissect out any potential interaction of [3H]-PIB with LB pathology. However, taking into account the absence of obvious labelling of LBs by [3H]-PIB in the three other PD cases, it seems probable that *in vivo* the cortical uptake of tracer associated with these lesions will be negligible in comparison with Aβ pathology.

## Discussion

Understanding the biological context of *in vivo* imaging data is of crucial importance for an accurate interpretation of the observed tracer uptake patterns. Research to date in the field of amyloid imaging has concentrated on understanding the interaction of these tracers with the Aβ pathology associated with AD. The present study focuses on AS filaments and provides a number of key findings directly applicable to imaging studies performed in dementia patient populations with concomitant LB pathology (Bacskai *et al.* 2007; Johansson *et al.* 2007; Rowe *et al.* 2007). It also significantly extends previous *in vitro* studies (Klunk *et al.* 2003; Fodero-Tavoletti *et al.* 2007).

Radioligand binding assays clearly identified the presence of a single class of binding sites on AS filaments to which the thioflavin S chemotypes (PIB, [3H]-Me-BTA-1 and BF1) all bound with a low nanomolar affinity. SB13 and FDDNP displayed substantially (> 20-fold) weaker binding to AS filaments. These findings clearly demonstrate that currently available ligands display a differential selectivity for the amyloid fold associated with *in vitro* generated AS filaments and provide the basis for a sensitive screening assay to develop AS-specific compounds. The results from this study extend and complement previous *in vitro* ligand binding analyses performed with AS filaments (Conway *et al.* 2000; Fodero-Tavoletti *et al.* 2007).

The study by Conway *et al.* (2000) demonstrated that filaments generated *in vitro* were both congophilic and able to enhance thioflavin T fluorescence but it did not provide a detailed analysis of the ligand binding sites. The recent study by Fodero-Tavoletti *et al.* (2007) investigated the binding of [3H]-PIB to *in vitro* generated AS filaments using a similar radioligand binding assay format to the one described here. However, in contrast to the present study, which found evidence for only a single site, their data analysis using Scatchard plots indicated the presence of two binding sites on AS filaments. The reasons for this discrepancy are unclear but may relate to differences in AS filament preparation, assay conditions and data analysis. Previously, we observed a tendency of Scatchard analyses to indicate the presence of multiple binding sites on Aβ fibrils that were not supported by analysis of the data using non-linear regression (Ye *et al.* 2006).

The interaction of a range of compounds, including previously characterised amyloid binding ligands, such as Me-BTA-1, with AS has also been reported using an assay format sensitive to potential inhibitors of filament formation (Masuda *et al.* 2006b). Although, the benzothiazole was found to be a weak inhibitor of filament assembly (IC_50_ > 80 μM) this does not preclude its high affinity interaction with the preformed AS filaments used in the current study. Furthermore, we found no effect on AS filament stability (as judged by ultracentrifugation) associated with any of the amyloid ligands following a 2 h incubation (data not shown).

The density of ligand binding sites was found to be one ligand binding site per ∼900 AS monomers. This is approximately threefold less than the density reported previously for *in vitro* generated Aβ fibrils (Lockhart *et al.* 2005) and may reflect a fundamental difference in the quaternary structure of the two polypeptide chains. Conceivably the observed reduction in binding site density could be achieved either through a simple reduction in the total number of binding sites or via steric blockade of a proportion of the available binding sites by regions of the AS monomer that are not incorporated in the backbone of the filament. Studies of AS filaments, either generated *in vitro* or isolated from brain of DLB patients, have shown that whilst the N-terminal region of AS is buried within the filament core, the C-terminal region is exposed on the filament surface (Crowther *et al.* 2000; Serpell *et al.* 2000; Chen *et al.* 2007). Fibre diffraction patterns of synthetic AS filament preparations have revealed the presence of a 0.47 nm-reflection at the meridian, characteristic of the cross-β sheet motif of amyloid (Serpell *et al.* 2000). These findings are consistent with the present observations showing that AS filaments can bind to amyloid-specific ligands such as PIB.

A limited exploration of the structural specificity of the radioligand binding site was performed through the use of displacement assays. However, further investigations into the presence of additional classes of binding sites similar to those associated with *in vitro* preparations of Aβ (Levine 2005; Lockhart *et al.* 2005; Ye *et al.* 2005) were not undertaken because of the relatively narrow range of ligand concentrations that could be explored, because of necessary omission of ethanol from the assays. The radioligand assays indicated that all of the compounds examined were able to displace the radiolabelled BTA derivative and that there were some potentially significant differences in the behaviour of the competitor ligands. The benzofuran derivative BF1 displayed both the highest affinity interaction (*K*_i_ = 4.78 nM) and the most consistent displacement, reflected in the low variance of the *K*_i_ values measured across different batches of AS. At the other end of the spectrum, FDDNP displayed the weakest binding to AS filaments (*K*_i_ = 210 nM) and the greatest variability across filament batches. The behaviour of the stilbene SB13 (*K*_i_ = 82 nM) fell somewhere in-between that of BF1 and FDDNP.

At first glance, this would suggest that SB13 and FDDNP are less selective for AS but, as discussed above, this difference may be overestimated because of the insolubility issues encountered when these highly lipophilic compounds were used at micromolar concentrations (Lockhart *et al.* 2005). The variability in *K*_i_ values may also suggest a degree of structural heterogeneity between batches of AS filaments. Different morphologies have previously been described for different batches of synthetic AS filaments (Heise *et al.* 2005; Kloepper *et al.* 2006). It is conceivable that as a result of its planar structure, FDDNP, the ligand which displayed the greatest variability, may be more sensitive to this variation. However, on balance the variability in *K*_i_ values can be more readily explained in terms of individual ligand solubility rather than potential heterogeneity in filament structure.

The radioligand binding data also provide an opportunity to compare ligand affinities between AS filaments and Aβ fibrils (Table 1). The BTA derivatives and BF1 displayed no apparent differences in their affinities for either polymer, consistent with very similar binding pockets associated with both amyloid filaments. Only FDDNP and SB13 presented large differences (> 5-fold) between AS and Aβ polymers, and although this might be because of differences in assay format, it nevertheless raises the possibility of engineering selectivity for the different neuropathological amyloid structures.

The autoradiography study clearly demonstrated that tracer concentrations of PIB did not bind to the extensive LB pathology associated with AMY sections. Although some caution must be observed, given the relatively small number of cases and brain regions examined, the data nevertheless indicate that *in vivo* LBs are unlikely to contribute significantly to the cortical uptake of PIB. These results significantly extend the previous, essentially histological, evaluation of PIB binding to LB pathology (Fodero-Tavoletti *et al.* 2007) which used fixed tissue, extensive tissue preparation techniques and fluorescence microscopy with a high concentration of ligand (100 μM). In contrast, the current study used fresh-frozen brain sections and minimal tissue preparation techniques in an effort to maintain the integrity of pathological features to as close as *in vivo* as possible. In addition radiotracer concentrations directly relevant to *in vivo* imaging were utilised and we believe that combined these factors significantly reduced the potential for a false, negative signal from this part of the study.

The lack of detectable binding of [3H]-PIB to the LB pathology contrasts with the high-affinity binding sites on *in vitro* generated AS filaments reported by the radioligand binding assays. However, as discussed above, the density of ligand binding sites associated with AS filaments was around threefold lower than that previously detected on synthetic Aβ fibrils (Klunk *et al.* 2003; Lockhart *et al.* 2005; Ye *et al.* 2005). It is conceivable that this lower concentration of binding sites is maintained *in vivo* on the AS filaments associated with LBs and that this, in turn, may lead to a low sensitivity of the tracer for this lesion. In contrast, the number of ligand binding sites associated with Aβ fibrils *in vivo* appears to be an order of magnitude higher than that found on fibrils produced *in vitro* (Klunk *et al.* 2003, 2005). Potential concerns over the ability of autoradiography to detect fine, microscopic lesions can be partly addressed, as an identical method was previously used to detect NFTs (Lockhart *et al.* 2007).

A number of factors may act to reduce the number of available *in vivo* binding sites; they include post-translational modifications of the AS and the presence of accessory proteins within the LBs. Although no study has addressed these issues so far (for any lesion) they could either chemically modify the binding site or simply produce steric hindrance to ligand binding. In LBs, at least some AS filaments are extensively phosphorylated and nitrated, in addition to being ubiquitinated (Goedert 2001; Kotzbauer *et al.* 2001). These modifications could in principle produce conformational changes within the filament structure.

In summary, this study provides the first detailed description of the interaction of imaging tracers with an amyloid pool other than fibrillar Aβ and additionally, complements the developing literature surrounding the application of amyloid agents in non-AD dementia patient populations such as DLB (Bacskai *et al.* 2007; Rowe *et al.* 2007). The present report provides comprehensive evidence indicating that although AS filaments possess binding sites compatible with current amyloid imaging probes, *in vivo* LBs are unlikely to represent a major source of cortical tracer uptake. In addition, this study provides further specific support to the observation that *in vivo* PIB is likely to be a non-disease specific imaging marker of Aβ peptide-related cerebral amyloidosis (Lockhart *et al.* 2007). Finally, the data suggest that the development of AS-specific probes, although feasible, will require significant investment in both biology and radiochemistry.

## Abbreviations used

AD: Alzheimer's disease
AMY: amygdala
AS: α-synuclein
Aβ: amyloid-beta peptide
BF1: 5-bromo-2-(4-dimethylaminophenyl)benzofuran
BTA-1: 2-(4′-methylaminophenyl)benzothiazole
DLB: dementia with Lewy bodies
FDDNP: 2-(1-{6-[(2-fluoroethyl(methyl)amino]-2-naphthyl}ethylidene)malononitrile
HSP: high-speed pellet
HSS: high-speed supernatant
LB: Lewy body
MAB: 3-[*N*-morpholino] propanesulphonic acid assay buffer
Me-BTA: 2-[4′-(methylamino)phenyl]-6-methylbenzothiazole
NFTs: neurofibrillary tangles
PBS: phosphate-buffered saline
PD: Parkinson's disease
PET: positron emission tomography
PIB: Pittsburgh Compound-B (2-[4′-(methylamino)phenyl]-6-hydroxybenzothiazole)
SB13: 4-*N*-methylamino-4′-hydroxystilbene
SDS: sodium dodecyl sulphate
SPs: senile plaques
